# Deficiency of peroxiredoxin 2 exacerbates angiotensin II-induced abdominal aortic aneurysm

**DOI:** 10.1038/s12276-020-00498-3

**Published:** 2020-09-14

**Authors:** Se-Jin Jeong, Min Ji Cho, Na Young Ko, Sinai Kim, In-Hyuk Jung, Jeong-Ki Min, Sang Hak Lee, Jong-Gil Park, Goo Taeg Oh

**Affiliations:** 1 Cardiovascular Division, Department of Medicine, Washington University School of Medicine, St. Louis, MO USA; 2 Biotherapeutics Translational Research Center, Korea Research Institute of Bioscience & Biotechnology, Daejeon, Republic of Korea; 3 Department of Biomolecular Science, University of Science & Technology (UST), Daejeon, Republic of Korea; 4 Immune and Vascular Cell Network Research Center, National Creative Initiatives, Department of Life Sciences, Ewha Womans University, Seoul, Republic of Korea; 5 Division of Cardiology, Department of Internal Medicine, Severance Hospital, Yonsei University College of Medicine, Seoul, Korea; 6 Cardiovascular Research Institute, Yonsei University College of Medicine, Seoul, Korea

**Keywords:** Aneurysm, Aneurysm

## Abstract

Abdominal aortic aneurysm (AAA) is an inflammatory vascular disease characterized by structural deterioration of the aorta caused by inflammation and oxidative stress, leading to aortic dilatation and rupture. Peroxiredoxin 2 (PRDX2), an antioxidant enzyme, has been reported as a potential negative regulator of inflammatory vascular diseases, and it has been identified as a protein that is increased in patients with ruptured AAA compared to patients with nonruptured AAA. In this study, we demonstrated that PRDX2 was a pivotal factor involved in the inhibition of AAA progression. PRDX2 levels were increased in AAA compared with those in normal aortas in both humans and mice. Ultrasound imaging revealed that the loss of PRDX2 accelerated the development of AAA in the early stages and increased AAA incidence in mice infused with angiotensin II (Ang II). *Prdx2*^*−/−*^ mice infused with Ang II exhibited increased aortic dilatation and maximal aortic diameter without a change in blood pressure. Structural deterioration of the aortas from *Prdx2*^*−/−*^ mice infused with Ang II was associated with increases in the degradation of elastin, oxidative stress, and intramural thrombi caused by microhemorrhages, immature neovessels, and the activation of matrix metalloproteinases compared to that observed in controls. Moreover, an increase in inflammatory responses, including the production of cell adhesion molecules and the accumulation of inflammatory cells and proinflammatory cytokines due to PRDX2 deficiency, accelerated Ang II-induced AAA progression. Our data confirm that PRDX2 plays a role as a negative regulator of the pathological process of AAA and suggest that increasing PRDX2 activity may be a novel strategy for the prevention and treatment of AAA.

## Abdominal aortic aneurysm: Potential enzyme biomarker identified

An enzyme with antioxidant properties may provide a biomarker and therapeutic agent to help treat abdominal aortic aneurysm (AAA). AAA involves the structural deterioration of the aorta through chronic inflammation and oxidative stress, and can trigger life-threatening artery rupture. An antioxidant enzyme called peroxiredoxin 2 (PRDX2) is increased in patients with ruptures, but whether its role in AAA is beneficial or detrimental is unclear. Goo Taeg Oh at the Ewha Womans University in Seoul, Jong-Gil Park at the Korea Research Institute of Bioscience and Biotechnology, Daejeon, South Korea, and co-workers examined the effect of PRDX2 on AAA progression. PRDX2 suppressed structural damage in mice, limiting artery dilation and protein degradation. Loss of PRDX2 accelerated AAA development. Measuring levels of PRDX2 may indicate AAA severity in patients, while boosting the enzyme could repair aortic damage.

## Introduction

Abdominal aortic aneurysm (AAA) is an aortic disorder with a prevalence of 2 to 8% in men older than 65 years. If rupture occurs, AAA has a mortality rate that has been reported to be between 85 and 90%^1,2^. Degradation of the elastic media, activation of various proteases, infiltration of inflammatory cells, increased production of cytokines and formation of neovessels are closely linked to the development of AAA^3^. Oxidative stress caused by reactive oxygen species (ROS) generation during inflammatory processes involved in the pathogenesis of AAA induces the activation of matrix metalloproteinases (MMPs) and proteolytic degradation of structural proteins^4^. Evidence of increased oxidative stress in tissue samples from patients with AAA implicates ROS in the pathogenesis of AAA^5–7^. Genetic and pharmacological inhibition of oxidative stress attenuates aneurysmal aortic diseases in animal models^8,9^. Despite our advanced understanding of AAA pathology, surgical management, including open surgery and endovascular repair, is currently the only proven effective intervention because of the lack of pharmacological agents^3,10^. Angiotensin-converting enzyme inhibitors and β blockers have failed to inhibit aneurysm expansion in clinical trials^11–13^. It is necessary to clarify the effects of statins, tetracycline, and macrolides on the progression of aneurysmal disease^14–18^. Thus, novel candidates for the regulation of AAA progression are needed for the development of pharmacological agents with favorable safety profiles.

Peroxiredoxins (PRDX) are a ubiquitous family of thiol-specific antioxidant enzymes that control the levels of intracellular peroxide, which is involved in oxidative stress and signal transduction^19–21^. In mammalian cells, the PRDX family includes six isoforms (PRDX1–PRDX6), which are distributed in various intracellular compartments^22^. PRDX1 and PRDX2 are the most abundant proteins among these six isoforms and exhibit high affinity for hydrogen peroxide at low concentrations in the cytosol of cells^23^. Recently, we demonstrated the anti-atherogenic roles of PRDX1 and PRDX2 in apolipoprotein E-deficient (*ApoE*^*−/−*^) mice in a specific manner^24,25^. PRDX1 plays an important role in lipophagic flux and maintains cholesterol homeostasis in macrophages in the presence of oxidative stress, leading to a decrease in the formation of foam cells and atherosclerotic plaques^24^. Deficiency of PRDX2 in *ApoE*^*−/−*^ mice enhanced the activation of p65, c-Jun, c-Jun N-terminal kinases, and p38 mitogen-activated protein kinase, accelerating the formation of atherosclerotic plaques^25^. PRDX2 deficiency in *ApoE*^*−/−*^ mice increased the expression of cell adhesion molecules (CAM) on vascular endothelial cells, which was followed by an increase in immune cell infiltration into the plaque^25^. Moreover, Choi et al.^26^ reported that PRDX2 negatively regulated platelet-derived growth factor (PDGF) receptor signaling and subsequently suppressed the proliferation and migration of vascular smooth muscle cells (VSMCs). In a study comparing the proteomic composition of aneurysmal aortas in patients with nonruptured aneurysms and those with ruptured aneurysms, higher levels of PRDX2 were revealed in patients with ruptured AAAs than in those with nonruptured AAAs^27^. However, the potential role of PRDX2 in the progression of AAA has not been explored. In this study, we revealed that PRDX2 inhibits the angiotensin II (Ang II)-induced formation of AAA by reducing oxidative stress and inflammatory responses in aortic lesions and preventing structural damage to the aorta.

## Materials and methods

### Human tissue studies

AAA tissue samples were obtained from patients during surgical repair of the aneurysmal aorta. Normal aortic tissues were obtained from the abdominal arteries of patients with end-stage renal disease during kidney transplantation. The samples were harvested from patients who underwent surgery at Severance Hospital, Yonsei University (Seoul, Republic of Korea). All patients provided written informed consent. To ensure the strict protection of privacy, identifying information was removed from all samples before analysis. This study conformed to the principles outlined in the Declaration of Helsinki and was approved by the Institutional Review Board of Severance Hospital (IRB No. 4-2013-0688).

### Generation of the mouse AAA model and animal studies

The *Prdx2*^*+/+*^ and *Prdx2*^*−/−*^ mice were derived from *C57BL/6J* congenic lines that were backcrossed more than 10 times with *C57BL/6J* mice. Mice were housed in polycarbonate cages (five per cage) under specific pathogen-free conditions and maintained under a 12-h light/dark cycle at 22–23 °C with ad libitum access to water. All studies were performed in accordance with the Guidelines on the Care and Use of Laboratory Animals (National Institutes of Health Publication no. 85–23, revised 1996) and were approved by the Institutional Animal Care and Usage Committee (IACUC No. 2012-01-052) of Ewha Womans University (Seoul, Republic of Korea). The formation of an aneurysm in the mouse model was induced through infusion of Ang II. Briefly, saline or Ang II was infused into 8-week-old mice via subcutaneous osmotic pumps at 1000 ng/kg/min for a maximum of 4 weeks. After the study, all animals were anesthetized by inhalation of isoflurane (3%) plus 1 L/min O_2_ and euthanized by exsanguination. The severity of the aneurysm (stage 0–4) was classified as previously described^28^.

### Ultrasound in vivo imaging

Echocardiography was performed under anesthesia through inhalation of 1.5–2% isoflurane (Forene isoflurane; Abbott Scandinavia AB, Kista, Sweden). Images were captured using the Vevo 2100 system (version 1.5.0; Visual Sonics, Toronto, ON, Canada) with a 30-MHz linear transducer. The maximal internal diameters of the aortic images were measured using VEVO 2100 software.

### Blood pressure

Blood pressure (BP) was measured using a noninvasive tail–cuff system (CODA-HT4; Kent Scientific Corporation, Torrington, CT). Briefly, mice were acclimated to BP measurement conditions on a daily basis for 1 week. After acclimation, BP was measured two times, once before and once after the implantation of the Ang II pumps, to determine the basal and Ang II-induced BP values in each mouse cohort. For the BP measurements, we placed the mice in tail–cuff restraints on a warmed surface (39 °C).

### Histological and immunofluorescence staining

For histological examination, hematoxylin and eosin (H&E) staining, and Movat pentachrome staining were performed. Anti-CD31 (MAB1398Z; Millipore, Burlington, MA), anti-alpha-smooth muscle actin (anti-α-SMA; A5228; Sigma-Aldrich, St. Louis, MO; and ab5694; Abcam, Cambridge, United Kingdom), anti-CD45 (Mab114; R&D Systems, Minneapolis, MN), anti-PRDX2 (ab109367; Abcam), anti-4-hydroxynonenal (anti-4-HNE; ab46545; Abcam), anti-DNA/RNA damage antibody (anti-8-OHG) (ab62623; Abcam), and anti-mouse macrophage/monocyte antibody (anti-MOMA2) (MCA519G; Bio-Rad, Hercules, CA) were used as primary antibodies for immunostaining. After incubation with the primary antibodies, Alexa 488 and 594 (Invitrogen, Carlsbad, CA) or biotinylated secondary antibodies with 3,3′-diaminobenzidine substrate (Vector Laboratories, Burlingame, CA) were used to visualize the antigens. Furthermore, 4′,6-diamidino-2-phenylindole (DAPI) or hematoxylin was used to label the nuclei. The negative control tissues were prepared in a similar manner using IgG isotype control antibodies (Santa Cruz Biotechnology, Dallas, TX). The immunofluorescence was imaged with an LSM 510 meta confocal microscope (Carl Zeiss, Oberkochen, Germany) or a BX53 microscope (Olympus, Tokyo, Japan).

### In situ zymography

We performed in situ zymography to evaluate the activity of MMP in atherosclerotic plaques. Freshly cut cryosections (10 μm thick) of the aortic sinus were incubated with a green fluorogenic gelatin substrate (DQ gelatin; Molecular Probes) according to the protocol provided by the manufacturer. MMP-mediated proteolysis was detected with an LSM 510 meta confocal microscope (Carl Zeiss), and MMP activity was measured using ZEN software (Carl Zeiss).

### In situ detection of ROS

Dihydroethidine (DHE) was used to visualize the production of superoxide. Briefly, aortic tissues (10 μm thick) were obtained from *Prdx2*^*+/+*^ and *Prdx2*^*−/−*^ mice infused with Ang II. Cross-sections of aortic tissues were incubated with DHE (10 μmol/L dissolved in dimethyl sulfoxide) in a light-protected humidified chamber at 37 °C for 15 min. The sections were not washed and were directly visualized using fluorescence microscopy (LSM 510 meta confocal microscope; Carl Zeiss).

### Isolation of mouse aortic VSMCs

Mouse aortic VSMCs were isolated from male mice (20–25 g) and maintained in Dulbecco’s modified Eagle medium containing 10% fetal bovine serum at 37 °C in a humidified atmosphere of 5% CO_2_ and 95% air, as described previously^29^. VSMCs were passaged four to six times at 70–80% confluence. Cells were treated with Ang II (100 nM) for 24 or 48 h.

### Immunoblotting and enzyme-linked immunosorbent assay

For the isolation of proteins, samples were lysed in radioimmunoprecipitation assay buffer (50 mM Tris-hydrochloride [pH 7.4], 150 mM sodium chloride, 0.1% sodium dodecyl sulfate, 1% NP-40, 0.25% sodium deoxycholate, 1 mM ethylenediaminetetraacetic acid, 1 mM sodium fluoride, and 1 mM sodium orthovanadate) and protease inhibitor cocktail (Roche Diagnostics, Basel, Switzerland). For the western blotting analysis, proteins were electrophoresed on 10% sodium dodecyl sulfate-polyacrylamide gels and transferred onto polyvinylidene difluoride membranes. The membranes were blocked with 4% skim milk in Tris-buffered saline containing 0.5% Tween-20. They were subsequently incubated with anti-PRDX2 (ab109367; Abcam), anti-β-actin (Abc-2004; Abclon, South Korea), anti-glyceraldehyde-3-phosphate dehydrogenase (anti-GAPDH; Santa Cruz Biotechnology), anti-hemoglobin (LS-BIO, LS-B12169), anti-vascular endothelial cadherin (anti-VE-Cadherin; AF1002; R&D Systems), anti-vascular endothelial growth factor receptor 2 (anti-VEGFR2; 9698; Cell Signaling Technology, Danvers, MA), anti-α-SMA (ab5694; Abcam), anti-MMP2 (AF1488; R&D Systems), anti-MMP9 (AF909; R&D Systems), anti-fibronectin (ab2413; Abcam), anti-collagen IV (ab6586; Abcam), anti-4-HNE (ab46545; Abcam), anti-intercellular adhesion molecule-1 (anti-ICAM-1; AF796; R&D Systems), anti-vascular CAM-1 (anti-VCAM-1; AF643; R&D Systems), and anti-CD45 (AF114; R&D Systems) primary antibodies. This was followed by incubation with horseradish peroxidase-conjugated secondary antibody (IgG). The immunoreactive bands were detected through enhanced chemiluminescence (Amersham). For the sandwich enzyme-linked immunosorbent assay, Duoset Ab pairs detecting interleukin (IL)-1β (DY401) and IL-6 (DY406) were purchased from R&D Systems.

### Two-dimensional gel electrophoresis and protein identification using matrix‐assisted laser desorption–ionization time of flight

A protein sample (1 mg) from each group (*n* = 3 pooled) was loaded and subjected to two-dimensional (2D) gel electrophoresis. The gels were stained with Coomassie dye and scanned using a photo scanner. The comparative analysis for the relative quantification of samples was performed using ImageMaster 2D Platinum7 (GE Healthcare, Waukesha, WI) to determine the differential expression of the proteins according to the protein spots. The mass of the dried peptide mix was analyzed using matrix-assisted laser desorption–ionization time-of-flight (MALDI‐TOF) mass spectrometry (ULTRAFLEX III; Bruker Daltonics, Billerica, MA). After digestion with trypsin, the dried peptide mix was resuspended in Tris acetate buffer and then mixed with α‐cyano‐4‐hydroxycinnamic acid (α‐CHCA, MW189.04 Da) matrix (sample-to-matrix ratio: 1:10). The resulting 2 µl sample was spotted onto the MALDI target plate. Further analysis was conducted using Flex Analysis software (Bruker) to obtain the peptide mass fingerprint of the individual peptide fragments. The resulting peptide masses were submitted to the MASCOT server (version 2.1; Matrix Science, Boston, MA).

### Statistical analysis

Values are expressed as percentages or the means ± standard error of the mean, as appropriate. The statistical tests included the two‐tailed Student’s *t* test, one-way analysis of variance and the Mann–Whitney *U* statistical test. *P* values <0.05 denoted statistical significance.

## Results

### Aneurysmal lesions increase the levels of PRDX2 in the aortas of humans and mice

**Fig. 1:**
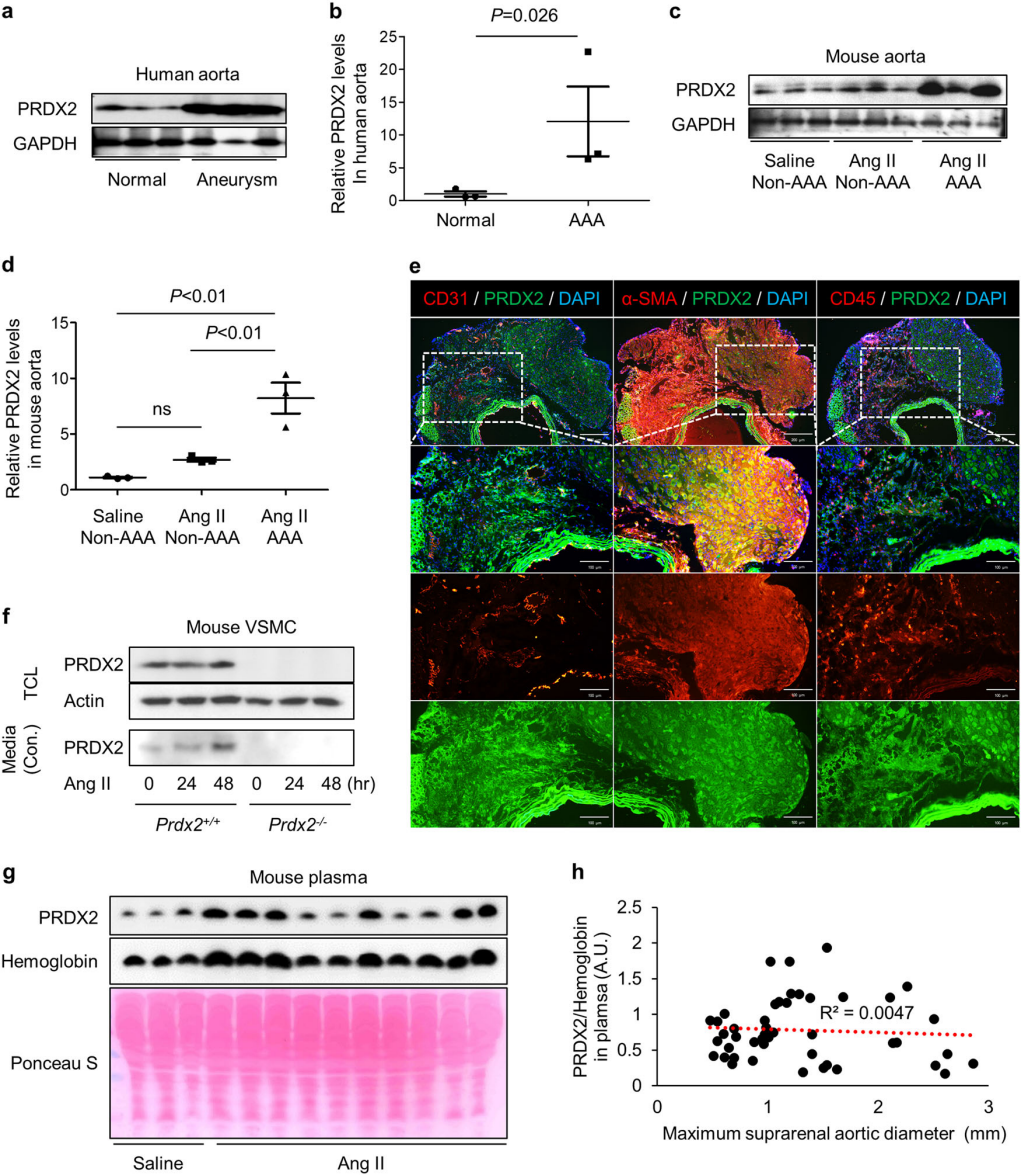
PRDX2 is increased in the aneurysmal aortas of humans and mice. **a** Immunoblotting analysis and **b** quantification of PRDX2 in healthy and aneurysmal aortas obtained from patients (*n* = 3). Data are presented as the mean ± SEM (two‐tailed Student’s *t* test). **c** Immunoblotting analysis and **d** quantification of PRDX2 in abdominal aortas obtained from *C57BL/6* mice infused with saline (non-AAA) and Ang II (non-AAA and AAA; *n* = 3). Data are presented as the mean ± SEM (one-way ANOVA). **e** Representative immunostaining images of PRDX2 (green) with CD31 (red, left), α-SMA (red, middle), or CD45 (red, right) on AAA. Cross-sectional low magnification images (top; scale bar, 200 µm) with higher magnification of the boxed area (lines 2–4; scale bar, 100 µm). Nuclei were stained with DAPI. **f** Immunoblotting analysis of PRDX2 in total cell lysates and concentrated culture media of primary VSMCs isolated from *Prdx2*^+/+^ and *Prdx2*^−/−^ mice. Ang II was administered to primary VSMCs during the indicated times. **g** Immunoblotting analysis of PRDX2 in plasma from mice infused with saline or Ang II. Hemoglobin was used for the normalization of PRDX2 levels in plasma obtained from RBC lysis. Ponceau S staining was used as a loading control. **h** Scatter plot and coefficient of determination between PRDX2/hemoglobin in mouse plasma and the maximum suprarenal aortic diameter of mice infused saline or Ang II.

Our approach was based on the significant changes in the composition of aneurysmal aortic tissues, which were characterized by intraluminal thrombus, immune cell infiltration, and alteration of VSMCs. We initially examined the expression of PRDX2 through immunoblotting analysis in normal or aneurysmal aortas from human patients and mice. Aneurysmal aortic lesions from patients with AAA expressed significantly higher levels of PRDX2 than those from normal controls (Fig. 1a, b). After infusion of saline or Ang II in 8-week-old *C57BL/6* male mice for 4 weeks via subcutaneous implantation of osmotic pumps, we isolated the suprarenal arteries, including normal aortas from saline-infused mice, non-AAA from Ang II-infused mice and AAA from Ang II-infused mice. The levels of PRDX2 were found to have increased dramatically in AAA from the Ang II group compared with those in normal aortas from the saline group or non-AAA from the Ang II group (Fig. 1c, d). Moreover, non-AAA from the Ang II group showed significantly higher expression of PRDX2 than normal aortas from the saline group (Fig. 1c, d). These results indicate that PRDX2 expression is closely linked to the development of AAA, suggesting the potential role of PRDX2 in AAA pathology in both humans and mice.

To explore the cellular localization of PRDX2 in AAA, we stained PRDX2 with CD31 to identify endothelial cells (ECs), α-SMA to identify VSMCs or CD45 to identify immune cells in AAA lesions. PRDX2 colocalized with markers of ECs, VSMCs, and immune cells in AAA lesions, but the levels of PRDX2 were higher in VSMCs than in other types of cells in AAA (Fig. 1e). Treatment of isolated primary VSMCs with Ang II slightly increased the expression of PRDX2 on day 2 and stimulated the release of PRDX2 into the media (Fig. 1f). Therefore, we tested whether the levels of PRDX2 could be detected in plasma from mice infused with saline or Ang II and whether they changed during the progression of AAA. The levels of PRDX2 in plasma were detected in both groups (Fig. 1g). As PRDX2 is one of the primary antioxidants in red blood cells (RBCs), we normalized the levels of PRDX2 in plasma according to the hemoglobin level. To evaluate the correlation between PRDX2/hemoglobin levels in plasma and the maximum suprarenal aortic diameter, we generated a scatter plot and determined the coefficient of determination. The coefficient of determination showed no significant correlation between the two factors (Fig. 1h and Supplementary Fig. 1).

### PRDX2 deficiency increases AAA incidence and aortic dilatation by Ang II

**Fig. 2:**
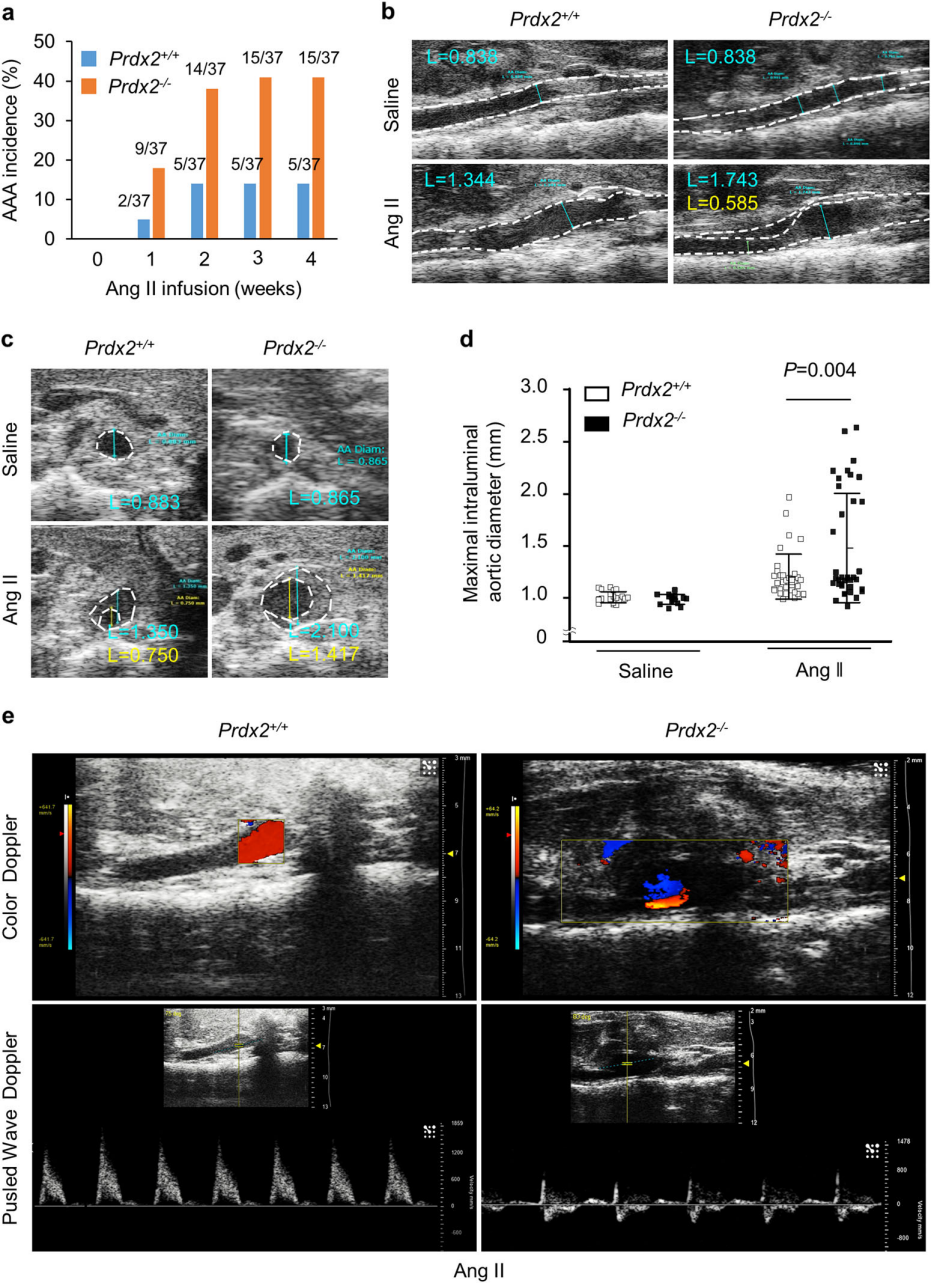
Loss of PRDX2 increases AAA incidence and aortic dilatation by Ang II. **a** The incidence of AAA (%) was evaluated using ultrasound imaging for 4 weeks in *Prdx2*^+/+^ and *Prdx2*^−/−^ mice infused with Ang II (*n* = 37). **b** Representative ultrasound imaging of the long axis and **c** the short axis of the suprarenal regions of *Prdx2*^+/+^ and *Prdx2*^−/−^ mice infused with saline or Ang II. **d** Quantification of the maximal intraluminal aortic diameter of the suprarenal regions from *Prdx2*^+/+^ and *Prdx2*^−/−^ mice infused with saline (*n* = 15–20) or Ang II (*n* = 37) by ultrasound imaging. Data are presented as the mean ± SEM (two‐tailed Student’s *t* test). **e** Representative ultrasound images of the long axis obtained from color Doppler (top) and pulse wave (bottom) mode in the suprarenal regions of *Prdx2*^+/+^ and *Prdx2*^−/−^ mice infused with Ang II.

To rule out the influence of other pathological factors, including hyperlipidemia and atherogenesis, on the progression of AAA, we used *C57BL/6* background mice to evaluate the effect of PRDX2 on the development of AAA. *Prdx2*^*+/+*^ and *Prdx2*^*−/−*^ male mice were subcutaneously infused with saline (*n* = 15–20) or Ang II (*n* = 37) for 4 weeks using osmotic pumps to determine the potential role of PRDX2 in the pathology of AAA. The development of AAA in mice was tracked using serial transabdominal ultrasound imaging. We found that the incidence of AAA was markedly different between *Prdx2*^*+/+*^ and *Prdx2*^*−/−*^ mice during Ang II infusion. *Prdx2*^*−/−*^ mice showed an increase in the incidence of AAA from 24% in week 1 to 40% in week 4. *Prdx2*^*+/+*^ mice showed an increase in the incidence of AAA from 5% in week 1 to 13% in week 4 (Fig. 2a). Ultrasound imaging in B-mode of the forward long and short axes revealed that the maximal intraluminal diameters in the suprarenal regions of the abdominal aorta were not different between *Prdx2*^*+/+*^ and *Prdx2*^*−/−*^ mice infused with saline. However, the loss of PRDX2 significantly increased the maximal intraluminal diameter in aortas from mice infused with Ang II in week 3 (Fig. 2b–d). In addition to increased aortic dilatation, we identified an increase in turbulent blood flow in the suprarenal regions of the abdominal aorta in *Prdx2*^*−/−*^ mice infused with Ang II compared with that in controls using color and pulsed wave Doppler (Fig. 2e). However, the two groups infused with saline showed normal blood flow in the suprarenal regions of the aorta (Supplementary Fig. 2).

### Loss of PRDX2 exacerbates Ang II-induced AAA without a change in BP

**Fig. 3:**
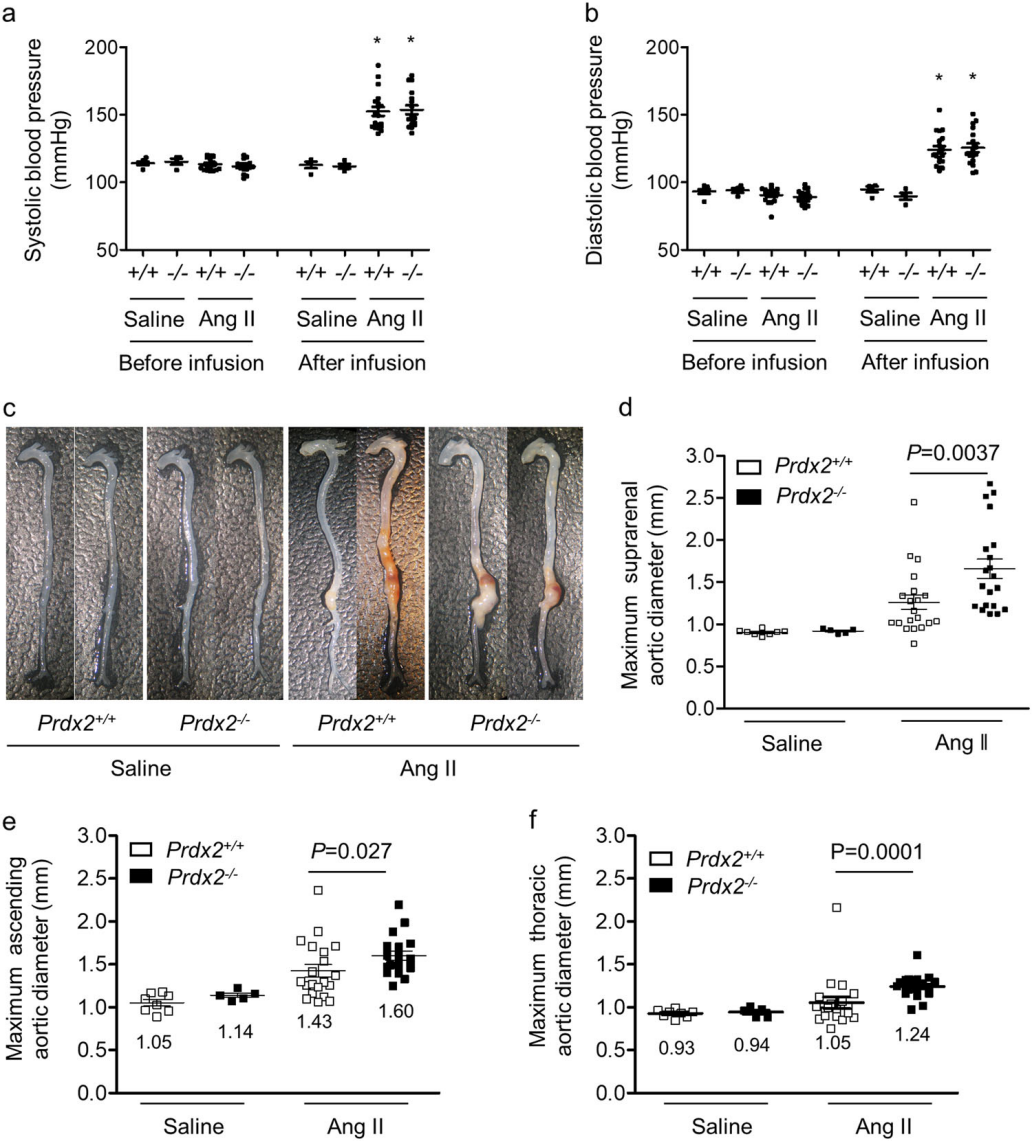
Deficiency of PRDX2 exacerbates Ang II-induced AAA without a change in blood pressure. **a** Systolic blood pressure in *Prdx2*^+/+^ and *Prdx2*^−/−^ mice infused with saline (*n* = 4–6) or Ang II (*n* = 17–18) at weeks 0 and 2. **b** Diastolic blood pressure in *Prdx2*^+/+^ and *Prdx2*^−/−^ mice infused with saline (*n* = 4–6) or Ang II (*n* = 17–18) at weeks 0 and 2. *P < 0.05 vs before infusion (two‐tailed Student’s *t* test for **a** and **b**). **c** Representative macroscopic images of whole aortas from *Prdx2*^+/+^ and *Prdx2*^−/−^ mice infused with saline or Ang II for 4 weeks. **d** Quantification of the maximal suprarenal aortic diameter, **e** the maximal ascending aortic diameter, and **f** the maximal thoracic aortic diameter in *Prdx2*^+/+^ and *Prdx2*^−/−^ mice infused with saline (*n* = 5–8) or Ang II (*n* = 20) for 4 weeks. All data are presented as the mean ± SEM (nonparametric Mann–Whitney *U* test for **d**–**f**).

An increase in BP is a risk factor for the formation of AAA^3^. Therefore, we tested the BP of mice in four groups using a noninvasive tail–cuff system. Infusion of Ang II in week 2 increased the systolic and diastolic BP in both groups compared with the BP values obtained before treatment. However, the loss of PRDX2 did not affect the susceptibility to Ang II-mediated alteration of BP (Fig. 3a, b). After 4 weeks, whole aortas from *Prdx2*^*+/+*^ and *Prdx2*^*−/−*^ mice infused with saline or Ang II were isolated and evaluated. Macroscopic images showed an increase in severe structural damage, including tortuous morphology and intramural thrombi, in the aortas of *Prdx2*^*−/−*^ mice infused with Ang II compared with that in aortas of *Prdx2*^*+/+*^ mice infused with Ang II (Fig. 3c). Moreover, the maximal aortic diameters in the suprarenal, ascending, and thoracic aortic regions were significantly enlarged in *Prdx2*^*−/−*^ mice infused with Ang II compared with those in *Prdx2*^*+/+*^ mice infused with Ang II. However, there was no difference between *Prdx2*^*+/+*^ and *Prdx2*^*−/−*^ mice infused with saline (Fig. 3d–f).

### Loss of PRDX2 disrupts the structural integrity of aortas in Ang II-induced AAA

**Fig. 4:**
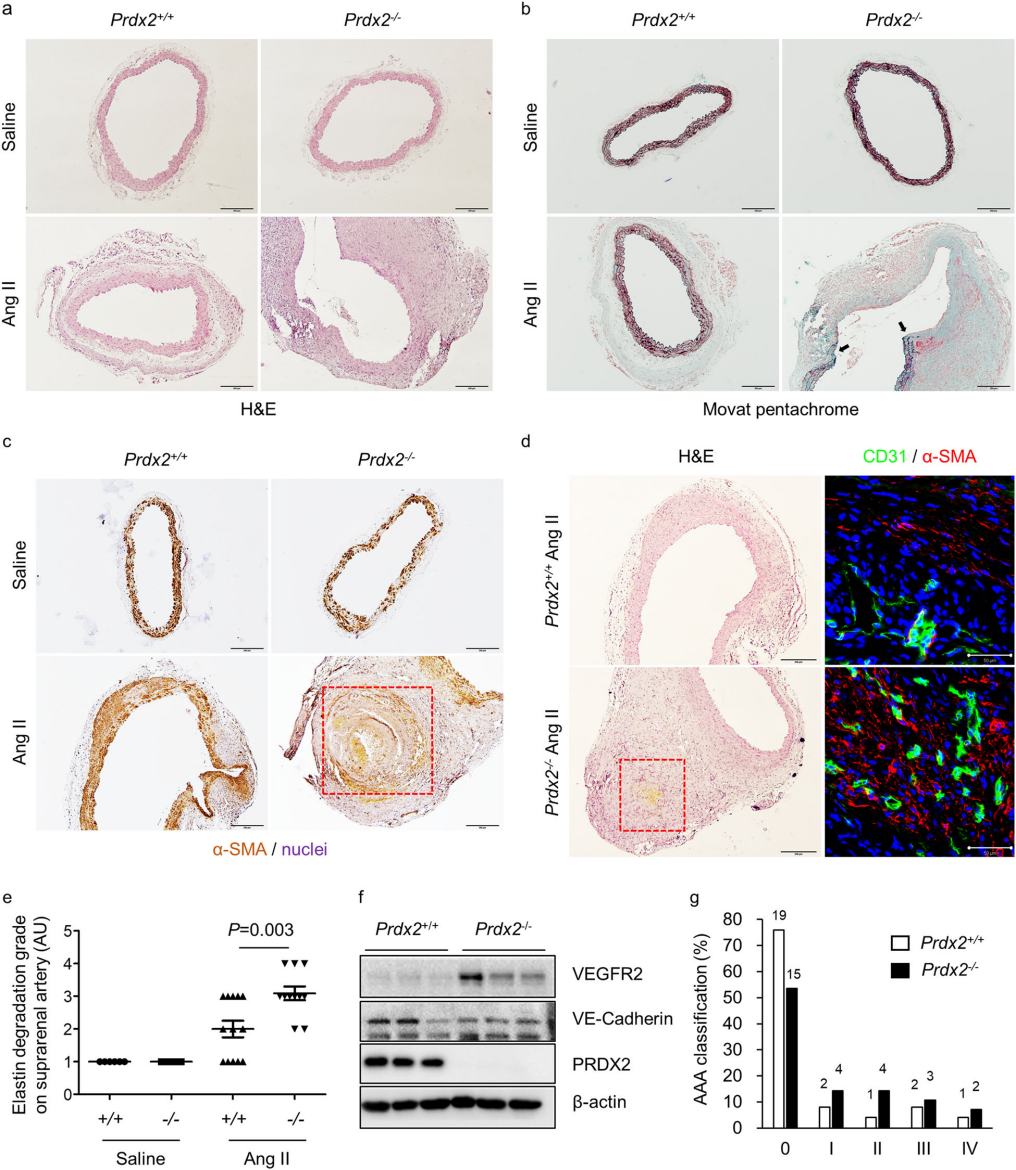
Loss of PRDX2 accelerates the structural deterioration of aortas in mice with Ang II-induced AAA. **a** Representative H&E- and **b** Movat pentachrome-stained images of cross-sectioned abdominal aortas from *Prdx2*^+/+^ and *Prdx2*^−/−^ mice infused with saline or Ang II for 4 weeks. Scale bar, 200 µm. **c** Representative immunostaining of α-SMA in cross-sectioned abdominal aortas. Nuclei were stained with hematoxylin. Scale bar, 200 µm. The boxed area indicates the apoptotic death of VSMCs. **d** Representative images of H&E (left; scale bar, 200 µm) staining and immunostaining of CD31 (green) and α-SMA (red) (right; scale bar, 50 µm) in cross-sectioned abdominal aortas. Nuclei were stained with DAPI. The boxed area shows microhemorrhage in the aneurysmal lesion. **e** Extent of elastin degradation in the suprarenal artery (AU) based on Movat pentachrome-stained images of abdominal aortic cross-sections (saline groups, *n* = 5–6; Ang II groups, *n* = 11–13). Data are presented as the mean ± SEM (two‐tailed Student’s *t*-test). **f** Immunoblotting analysis of VE-cadherin, VEGFR2, and PRDX2 in aneurysmal aortas from *Prdx2*^+/+^ and *Prdx2*^−/−^ mice infused with Ang II for 4 weeks. β-actin was used as a loading control. **g** AAA classification (%) of *Prdx2*^+/+^ (*n* = 25) and *Prdx2*^−/−^ (*n* = 28) mice infused with Ang II for 4 weeks according to a scale from 0 (no aneurysm) to 4 (multiple aneurysms with thrombi).

Subsequently, we evaluated the severity of AAA by characterizing the abdominal aortic lesions. According to Daugherty’s classification of AAA^28^, the abdominal aortic lesions of *Prdx2*^*+/+*^ and *Prdx2*^*−/−*^ mice infused with Ang II were classified from 0 (no aneurysm) to 4 (multiple aneurysms with thrombi) using macroscopic images and histological analysis with H&E and Movat pentachrome staining of aortic cross-sections. After cross-sectional dissection of AAA regions, we found increased dilatation and severe structural disruption in the abdominal aortic lesions of *Prdx2*^*−/−*^ mice infused with Ang II compared with those of controls (Fig. 4a). Additionally, Ang II-induced degradation of the elastic lamina in the suprarenal artery was significantly increased in PRDX2-deficient mice versus that in controls, whereas there was no difference in the groups infused with saline (Fig. 4b, e and Supplementary Fig. 3a). Using α-SMA immunostaining, we identified that the expansion of VSMCs in the aneurysmal lesion was elevated in *Prdx2*^*−/−*^ mice infused with Ang II compared with that in controls (Fig. 4c and Supplementary Fig. 3b). Moreover, α-SMA immunostaining demonstrated that AAA lesions from *Prdx2*^*−/−*^ mice infused with Ang II contained a larger area of apoptotic VSMC death than those from controls (Fig. 4c and Supplementary Fig. 3c). With the increase in intramural thrombi, as shown in Fig. 3c, the loss of PRDX2 locally caused an increase in microhemorrhage in aneurysmal lesions (Fig. 4d). After performing 2D gel electrophoresis of proteins from aortas of *Prdx2*^*+/+*^ and *Prdx2*^*−/−*^ mice infused with Ang II, we identified 37 increased spots and 23 decreased spots with differences of at least twofold between the two groups using MALDI-TOF analysis (Supplementary Fig. 4a, b), which revealed that albumin precursor levels were significantly increased in AAA from *Prdx2*^*−/−*^ mice infused with Ang II compared with those from controls (Supplementary Table 1). The increase in angiogenic ECs in AAA from *Prdx2*^*−/−*^ mice infused with Ang II was confirmed by immunostaining for CD31 in aneurysmal lesions (Fig. 4d) and by immunoblotting of VEGFR2 in aneurysmal aortas (Fig. 4f). However, coimmunostaining for CD31 and α-SMA in aneurysmal lesions revealed that the excess angiogenic ECs were not covered by VSMCs (Fig. 4d). In addition, the levels of VE-cadherin, which contributes to the integrity of EC-EC junctions, were reduced in the AAA of *Prdx2*^*−/−*^ mice infused with Ang II compared with those of controls (Fig. 4f). According to macroscopic images and histological analysis, we classified AAA from *Prdx2*^*+/+*^ (*n* = 25) and *Prdx2*^*−/−*^ (*n* = 28) mice infused with Ang II. In all stages (except stage 0), the loss of PRDX2 caused an increase in the incidence of AAA in mice infused with Ang II (Fig. 4g). These results demonstrated that the loss of PRDX2 was crucially involved in the expansion and vulnerability of aneurysmal aortas.

### Loss of PRDX2 increases the activity of MMPs and oxidative stress in Ang II-induced AAA

**Fig. 5:**
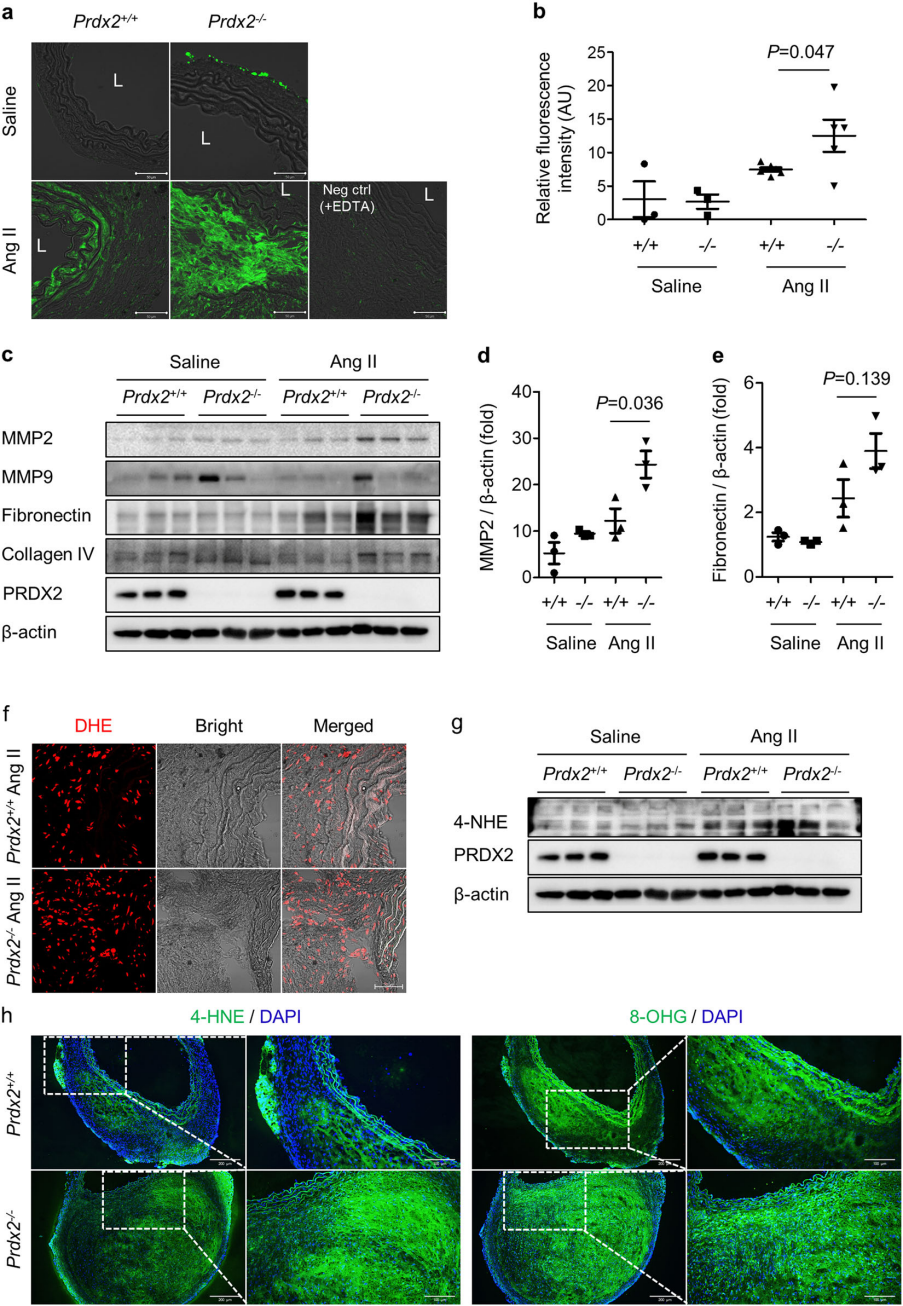
Deficiency of PRDX2 augments the activity of MMPs and oxidative stress in Ang II-induced AAA. Analysis of abdominal aortas from *Prdx2*^+/+^ and *Prdx2*^−/−^ mice infused with saline or Ang II for 4 weeks. **a** Representative in situ zymography images and **b** quantification using cross-sectioned abdominal aortas (saline groups, *n* = 3; Ang II groups, *n* = 5–6). Scale bar, 50 µm. Data are presented as the mean ± SEM (two‐tailed Student’s *t* test). **c** Immunoblotting analysis of MMP2, MMP9, fibronectin, collagen IV, and PRDX2 in aortas from *Prdx2*^+/+^ and *Prdx2*^−/−^ mice infused with saline or Ang II for 4 weeks. β-actin was used as a loading control. **d** Quantification of MMP2 and **e** fibronectin in Fig 5**c**. Data are presented as the mean ± SEM (two‐tailed Student’s *t* test). **f** Representative confocal images of DHE staining in abdominal aortic cross-sections. Scale bar, 50 µm. **g** Immunoblotting analysis of 4-HNE in aortas from *Prdx2*^+/+^ and *Prdx2*^−/−^ mice infused with saline or Ang II for 4 weeks. β-actin was used as a loading control. **h** Representative immunostaining images of 4-HNE (left) and 8-OHG (right) in abdominal aortic cross-sections. Low magnification images (scale bar, 200 µm) and higher magnification of the boxed area (scale bar, 100 µm). Nuclei were stained with DAPI.

MMP activity is closely associated with vascular remodeling and changes in the structural integrity of aortas in Ang II-induced AAA formation^4^. Thus, we measured MMP activity in aneurysmal aortic lesions using in situ zymography, which revealed an increase in fluorescence intensity in *Prdx2*^*−/−*^ mice infused with Ang II versus that in *Prdx2*^*+/+*^ mice (Fig. 5a, b and Supplementary Fig. 5a). AAA from *Prdx2*^*−/−*^ mice infused with Ang II showed an increase in MMP2 levels but not MMP9 levels compared with those from controls according to immunoblotting (Fig. 5c, d). Extracellular matrix components, including fibronectin and collagen IV, were more abundant in aneurysmal lesions from *Prdx2*^*−/−*^ mice infused with Ang II than in those from controls (Fig. 5c, e), suggesting that there was an increase in vascular remodeling caused by the loss of PRDX2. By using a proteomics approach, it was shown that the levels of heat shock proteins (HSPs), including HSP90, HSP70, and HSP60, were also significantly increased in aneurysmal aortas from *Prdx2*^*−/−*^ mice compared with those from controls (Supplementary Table 1), indicating that the loss of PRDX2 induced stressful conditions, including aortic tissue wounding and remodeling. These data suggested that the loss of PRDX2 accelerated the structural damage of aortas in mice after the administration of Ang II, leading to dilatation and structural deterioration of the aortas.

ROS have been considered a crucial stimulator for the activation of MMPs^30–32^. To evaluate oxidative stress in aneurysmal tissues from *Prdx2*^*+/+*^ and *Prdx2*^*−/−*^ mice infused with Ang II, we detected superoxide and hydrogen peroxide using DHE and DCF-DA staining, respectively. DHE staining revealed increased superoxide levels in aneurysmal aorta lesions in *Prdx2*^*−/−*^ mice infused with Ang II compared with those in controls (Fig. 5f), but we failed to detect the levels of hydrogen peroxide in the lesions (data not shown). Therefore, we tried to measure other oxidative stress indicators, such as the levels of 4-HNE and 8-OHG^33,34^. The levels of 4-HNE were increased in AAA from the Ang II-infused groups compared with those in normal aortas from the saline groups (Fig. 5g). Moreover, AAA from Ang II-infused *Prdx2*^*−/−*^ mice showed significantly higher levels of 4-HNE than AAA from Ang II-infused *Prdx2*^*+/+*^ mice (Fig. 5g and Supplementary Fig. 5b). Moreover, immunostaining of 4-HNE and 8-OHG revealed that the loss of PRDX2 augmented oxidative stress during the development of AAA (Fig. 5h). An increase in the level of succinate dehydrogenase complex subunit A, which is involved in ROS formation^35^, also indicated the increase in ROS in aortas after the loss of PRDX2 (Supplementary Table 1). Taken together, the results indicated that the loss of PRDX2 in mice augmented the oxidative stress level in aneurysmal lesions induced by Ang II infusion.

### PRDX2 prevents inflammation in aneurysmal aortic lesions

**Fig. 6:**
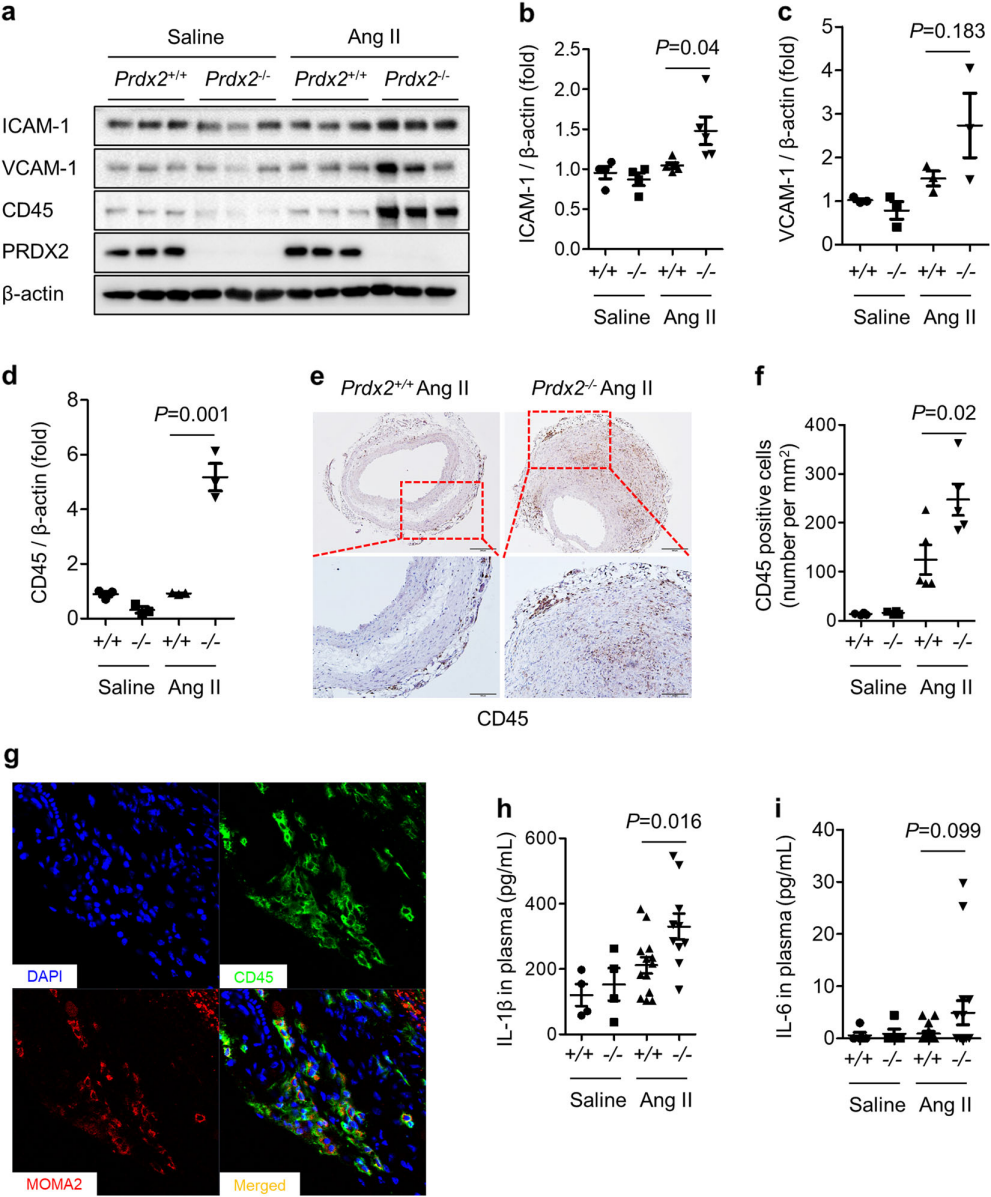
Loss of PRDX2 exacerbates inflammation in aneurysmal lesions. Analysis of abdominal aortas from *Prdx2*^+/+^ and *Prdx2*^−/−^ mice infused with saline or Ang II for 4 weeks. **a** Immunoblotting analysis of ICAM-1, VCAM-1, CD45, and PRDX2 in aortas from *Prdx2*^+/+^ and *Prdx2*^−/−^ mice infused with saline or Ang II for 4 weeks. β-actin was used as a loading control. **b** Quantification of ICAM-1, **c** VCAM-1, and **d** CD45 in Fig. 6a. Data are presented as the mean ± SEM (two‐tailed Student’s *t* test). **e** Representative immunostaining images showing the accumulation of immune cells in aneurysmal lesions. Cross-sectional images of CD45 immunostaining (top; scale bar, 200 µm) and higher magnification of the boxed area (bottom; scale bar, 100 µm). **f** Quantification of CD45-positive cells using images of abdominal aortic cross-sections (saline groups, *n* = 3; Ang II groups, *n* = 5). Data are presented as the mean ± SEM (two‐tailed Student’s *t* test). **g** Representative confocal images of immune cells (CD45) and macrophages (MOMA2) in aneurysmal aortas. Scale bar, 50 µm. **h** The levels of IL-1β and **i** IL-6 in plasma from *Prdx2*^+/+^ and *Prdx2*^−/−^ mice infused with saline or Ang II for 4 weeks. Data are presented as the mean ± SEM (two‐tailed Student’s *t* test).

Next, we tested whether the loss of PRDX2 regulates inflammation during the pathogenesis of AAA. Our previous study showed that PRDX2 deficiency in *ApoE*^*−/−*^ mice induced the expression of CAMs such as intercellular adhesion molecule-1 (ICAM-1) and vascular CAM-1 (VCAM-1) during the development of atherosclerosis^25^. Therefore, we first verified the levels of ICAM-1 and VCAM-1 in the aortas from *Prdx2*^*+/+*^ and *Prdx2*^*−/−*^ mice infused with saline or Ang II. ICAM-1 levels were more highly increased in the aortas from *Prdx2*^*−/−*^ mice infused with Ang II than those in aortas from controls (Fig. 6a, b). VCAM-1 expression showed a higher increase with the loss of PRDX2 expression in Ang II-infused mice than in controls, but this was not significant (Fig. 6a, c). Consistent with the increase in CAMs, the levels of CD45, which serves as a marker of immune cells, were markedly increased in AAA from *Prdx2*^*−/−*^ mice infused with Ang II compared with those in AAA from the controls (Fig. 6a, d). Moreover, we examined the extent of immune cell infiltration in the aneurysmal aortic lesions using CD45 immunostaining and found that it was significantly increased in *Prdx2*^*−/−*^ mice infused with Ang II versus that in *Prdx2*^*+/+*^ mice (Fig. 6e, f). MOMA2 immunostaining revealed that most CD45-positive cells in aneurysmal lesions were macrophages (Fig. 6g). Interestingly, PRDX2 deficiency systemically induced inflammation, which was in line with the increase in IL-1β and IL-6 levels in the plasma of *Prdx2*^*−/−*^ mice infused with Ang II versus those in *Prdx2*^*+/+*^ mice (Fig. 6h, i). In the proteomic analysis, an increase in inflammation-related proteins, including leukotriene A-4 hydrolase, H-2 class I histocompatibility antigen, and TLA(B) alpha chain-like, was also revealed in *Prdx2*^*−/−*^ mice infused with Ang II (Supplementary Table 1). These results demonstrated that PRDX2 deficiency accelerated inflammation in ECs and immune cells during the progression of AAA.

## Discussion

**Fig. 7:**
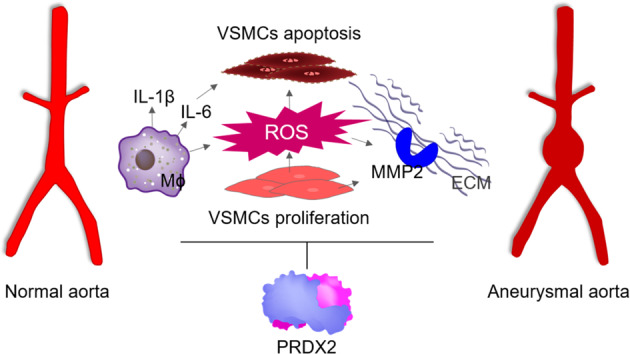
PRDX2 prevents structural damage, oxidative stress, and inflammation during the progression of AAA. The levels of PRDX2 are augmented in aneurysmal aortas and participate in ameliorating the structural deterioration of aortas, ROS-mediated oxidative stress, and inflammatory responses induced by Ang II administration.

Our results demonstrate the suppressive effects of PRDX2 on the progression of AAA via the inhibition of structural damage, oxidative stress, and inflammatory responses in AAA lesions. We characterized four ways in which PRDX2 ameliorates AAA progression (Fig. 7). First, the expression of PRDX2 is markedly increased in the aneurysmal aortas from both human patients and the Ang II-induced mouse model versus that in aortas from normal controls. Second, PRDX2 prevents the Ang II-induced structural deterioration of aortas, including elastin degradation and aortic dilatation. Third, PRDX2 alleviates the activation of MMPs and reduces oxidative stress in Ang II-induced AAA formation. Finally, the anti-inflammatory properties of PRDX2 inhibit the expression of CAMs and the accumulation of CD45-positive immune cells in AAA. Therefore, we proposed the inhibitory role of PRDX2 in AAA progression and other vascular diseases involving oxidative stress and inflammation.

A previous study reported increased levels of PRDX2 in ruptured aortic aneurysm tissue versus those in nonruptured aortic aneurysm tissue^27^. In this study, we demonstrated an increase in PRDX2 levels in the abdominal aortas of patients with AAA versus that in healthy donors. Moreover, aneurysmal aortas from Ang II-infused mice also showed an increase in PRDX2 similar to that found in human aneurysmal aortas versus that in controls. Intraluminal and intramural thrombi of aneurysmal aortas may enhance the levels of PRDX2 because PRDX2 is the primary antioxidant in RBCs^36^. Moreover, VSMCs treated with Ang II showed a slight increase in PRDX2 levels, which may affect the increase in PRDX2 after VSMC proliferation in Ang II-induced aneurysmal aortas. With regard to the cellular expression of PRDX2, we have reported that PRDX2 is expressed in vascular cells, including ECs and VSMCs, and macrophages derived from the peritoneal cavity and bone marrow^24^. As was the case in our previous data, we confirmed that PRDX2 expression was higher in VSMCs than in ECs and immune cells using immunostaining.

Mullen et al.^37^ reported that PRDX1 and PRDX2, which do not have signal peptides, are secreted from various cells in response to inflammatory stimuli through exosomal release. Martinen-Pinna et al.^38^ reported that PRDX1, which is mainly released from RBCs and polymorphonuclear neutrophils localized to the luminal part of the AAA thrombus, was increased in the serum of patients with AAA compared with that in the serum of healthy individuals. This result was in line with the positive correlation of the diameter of the AAA and the expansion rate. Then, we tried to determine whether the levels of PRDX2 in plasma are correlated with AAA progression. Ang II-infused mice showed a greater increase in plasma PRDX2 than saline-infused mice. However, the levels of hemoglobin in plasma were also comparable with the PRDX2 levels, suggesting that contamination by RBC lysates may play a role in the increase in plasma PRDX2. Therefore, the increase in plasma PRDX2 could be meaningful as a biomarker for the determination of AAA formation or severity if luminal thrombi and RBC lysis are involved in the pathological process of AAA.

ROS are locally increased in AAA and contribute to the pathogenesis of aortic aneurysms in both human and animal models^5,7,39^. ROS production in the vasculature has been associated with inflammation and the structural integrity of aortas. Because PRDX2 is a cellular peroxidase that removes intracellular hydrogen peroxides^19^, we attempted to show the levels of hydrogen peroxides in AAA lesions but failed because of technical issues. However, we confirmed the increase in oxidative stress in AAA lesions from PRDX2-deficient mice by DHE staining, 4-HNE and 8-OHG immunostaining, and 4-HNE immunoblotting analysis. Infiltrating inflammatory cells in the vasculature produce ROS and proinflammatory cytokines, leading to the production of ROS by VSMCs^40,41^. Moreover, elevated levels of ROS promote aortic dilatation and rupture through the MMP-mediated degradation of extracellular matrix and apoptosis of VSMCs^40–43^. Using ultrasound imaging and histological analysis, we demonstrated that the loss of PRDX2 induced an increase in AAA incidence along with aortic dilatation and the structural warping of aortas following an increase in the infiltration of inflammatory cells into lesions and MMP activation. Because macrophage-derived MMP9 and mesenchymal cell-derived MMP2 are both important for the development of aortic aneurysms^44^, we tested the levels of MMP2 and MMP9 in AAA from *Prdx2*^*+/+*^ and *Prdx2*^*−/−*^ mice infused with saline or Ang II. Immunoblotting analysis revealed an increase in MMP2 levels in AAA from Ang II-infused *Prdx2*^*−/−*^ mice, revealing that VSMC-derived MMP2 may be critical for MMP-mediated extracellular degradation in PRDX2-deficient mice. Moreover, the infiltration of immune cells induces damage to aortic tissues through the release of proinflammatory cytokines, including IL-1β and IL-6, which promote the apoptotic death of VSMCs and aortic dilatation^45,46^. In this study, we identified that the levels of IL-1β and IL-6 in plasma from *Prdx2*^*−/−*^ mice infused with Ang II were increased compared with those in plasma from control mice. However, tumor necrosis factor-α was not detectable in plasma from all groups, and the levels of monocyte chemoattractant protein-1 were comparable in plasma from *Prdx2*^*+/+*^ and *Prdx2*^*−/−*^ mice infused with Ang II (data not shown).

PRDX2 has been considered a regulator of cellular signaling via its control of endogenous hydrogen peroxides produced by various stimuli, including PDGF^26^. PDGF, which induces the generation of hydrogen peroxides for PDGF receptor signal transduction, is a potent growth factor that leads to the proliferation and migration of VSMCs^47^. Choi et al.^26^ reported that the loss of PRDX2 caused the elevation of endogenous hydrogen peroxides, leading to the proliferation and migration of VSMCs through the strengthening of PDGF receptor signaling. Ang II has multiple physiological effects, including the regulation of cell proliferation and migration via the regulation of various growth factors such as PDGF. VSMC proliferation and migration are among the features of the pathophysiological process of AAA formation^48^. Loss of PRDX2 induced the proliferation and migration of VSMCs during the development of AAA, leading to an increase in the maximum aortic diameter. We verified that multiple treatments rather than the single treatment of VSMCs with Ang II amplified proliferation, which likely occurred via PDGF (data not shown). However, it must be confirmed whether the loss of PRDX2 in VSMCs accelerates proliferation and migration caused by treatment with Ang II. Moreover, Ang II modulates VEGF-driven angiogenesis in a specific manner via type 1 and type 2 receptors. In this study, we provided evidence that the loss of PRDX2 was associated with the formation of immature neovessels in AAA lesions. Kang et al.^49^ reported that PRDX2 prevents the oxidative inactivation of VEGFR2 in ECs, which partly disagrees with our data. Ang II-induced angiogenesis could be mediated by multiple factors, including ROS and angiopoietin-2^50^. Additionally, the use of different disease models might explain the discrepancy between the two studies. Finally, VSMC proliferation and migration may lead to the expansion of aneurysmal aortas and increases in the apoptotic death of VSMCs due to immature angiogenesis, oxidative stress and inflammation, leading to AAA progression and structural vulnerability in aortas in PRDX2-deficient mice.

In summary, the loss of PRDX2 accelerated the progression of AAA pathophysiology through an increase in inflammatory responses and structural damage to the aorta. Although there is a need to examine the potential therapeutic role of PRDX2 in AAA, our data support the importance of PRDX2 in the development of aneurysmal diseases. Moreover, the increased levels of PRDX2 in human aneurysmal tissues or plasma may provide an approach for developing a potential biomarker to track the pathological development of AAA.

## Supplementary information

Supplemental material
