# Supplementary material for: Deficiency of peroxiredoxin 2 exacerbates angiotensin II-induced abdominal aortic aneurysm
Source: Exp Mol Med. 2020 Sep 14;52(9):1587–601. doi: 10.1038/s12276-020-00498-3 (PMC8080566; doi:10.1038/s12276-020-00498-3)
Supplement: Supplementary file 1 — Supplemental material [file 12276_2020_498_MOESM1_ESM.docx]

**SUPPLEMENTARY INFORMATION**

**Deficiency of Peroxiredoxin 2 Exacerbates Angiotensin II-induced Abdominal Aortic Aneurysm**

**Short title**: Role of Peroxiredoxin 2 on Aneurysm

Se-Jin Jeong^1,*^, Min Ji Cho^2,3,*^, Na Young Ko^4^, Sinai Kim^4^, In-Hyuk Jung^1^, Jeong-Ki Min^2,3^, Sang Hak Lee^5,6^, Jong-Gil Park^2^, Goo Taeg Oh^4^

^1^ Cardiovascular Division, Department of Medicine, Washington University School of Medicine, St. Louis, MO, USA

^2^ Biotherapeutics Translational Research Center, Korea Research Institute of Bioscience & Biotechnology, Daejeon, Republic of Korea

^3^ Department of Biomolecular Science, University of Science & Technology (UST), Daejeon, Republic of Korea

^4^ Immune and Vascular Cell Network Research Center, National Creative Initiatives, Department of Life Sciences, Ewha Womans University, Seoul, Republic of Korea

^5^ Division of Cardiology, Department of Internal Medicine, Severance Hospital, Yonsei University College of Medicine, Seoul, Korea

^6^ Cardiovascular Research Institute, Yonsei University College of Medicine, Seoul, Korea

^*^ These authors contributed equally to this work.

Correspondence to Goo Taeg Oh, DVM, PhD, Department of Life Sciences, Ewha Womans University, 52 Ewhayeodae-gil, Seodaemun-gu, Seoul, 120-750, Republic of Korea. Tel: +82-2-3277-4128; Fax: +82-2-3277-3760; E-mail: [gootaeg@ewha.ac.kr](mailto:gootaeg@ewha.ac.kr)

Correspondence to Jong-Gil Park, PhD, Biotherapeutics Translational Research Center, Korea Research Institute of Bioscience & Biotechnology, 125 Gwahak-ro, Yuseong-gu, Daejeon 34141, Republic of Korea. Tel: +82-42-860-4122; Fax: +82-42-860-4149; E-mail: [jonggilpark@kribb.re.kr](mailto:jonggilpark@kribb.re.kr)

**Supplementary figure legends**

**Supplementary Fig. 1.** Immunoblotting analysis of PRDX2 in plasmas from mice infused with saline or Ang II. Haemoglobin was used for the normalisation of PRDX2 levels in plasmas from RBCs lysis. Ponceau S staining was used as a loading control. Arrow heads indicate the same sample, which were loaded for comparison among the different immunoblotting analysis.

**Supplementary Fig. 2.** Representative ultrasound images using the long axis on colour Doppler (top) and pulse wave (bottom) mode in the suprarenal regions of *Prdx2^+/+^* and *Prdx2^−/−^* mice infused with saline.

**Supplementary Fig. 3.** (a) Representative images of autofluorescence for elastin on cross-sectional abdominal aortas from *Prdx2^+/+^* and *Prdx2^−/−^* mice infused with Ang II. Nuclei were stained with DAPI. Scale bar, 200 µm. (b) Representative immunostaining images of α-SMA (red) on cross-sectional abdominal aortas from *Prdx2^+/+^* and *Prdx2^−/−^* mice infused with Ang II. Boxed area indicates proliferated VSMCs in AAA lesions. Nuclei were stained with DAPI. Scale bar, 200 µm. (c) Representative images of acellular regions on cross-sectional abdominal aortas from *Prdx2^+/+^* and *Prdx2^−/−^* mice infused with Ang II. Nuclei were stained with DAPI. Scale bar, 200 µm.

**Supplementary Fig. 4.** Proteomic analysis using 2D gel electrophoresis and MALDI-TOF mass spectrometry using aortas of *Prdx2^+/+^* and *Prdx2^−/−^* mice infused with Ang II for 4 weeks. (a) Images for 2D gel electrophoresis. A total of 716 and 704 spots were detected in aortas from *Prdx2^+/+^* and *Prdx2^−/−^* mice infused with Ang II, respectively. Green indicated paired spots, and red indicated non-paired spots between the two groups. (b) Pie charts showing the significantly increased proteins (top) and significantly decreased proteins (bottom) in the aortas of *Prdx2^−/−^* mice infused with Ang II versus controls.

**Supplementary Fig. 5.** (a) Plots depict 2D graphical reconstructions of the green intensity profile of *in situ* zymography images in Figure 5A. (b) Quantification of 4-HNE in Figure 5G. Data are presented as the mean ± SEM (two‐tailed Student’s *t* test).

**Table 1.** Increased proteins in the proteomic analysis through 2D gel electrophoresis and MALDI-TOF mass spectrometry in aneurysmal aortas from *Prdx2^−/−^* mice infused with Ang II versus controls.

| Group ID | MS Identification | Mascot score | Prdx2^+/+^ AngII % vol. | Prdx2^-/-^ AngII % vol. | Prdx2^-/-^ AngII / Prdx2^+/+^ AngII Ratio |
| --- | --- | --- | --- | --- | --- |
| 77 | collagen alpha-1(VI) chain precursor | 138 | 0.047 | 0.102 | 2.2 |
| 111 | 2-oxoglutarate dehydrogenase (mitochondrial precursor), oxoglutarate dehydrogenase (lipoamide) | 157 | 0.026 | 0.052 | 2.0 |
| 134 | endoplasmin, Heat shock protein 90, beta (Grp94), member 1 | 221 | 0.089 | 0.200 | 2.2 |
| 138 | mKIAA0172 protein, Kank1 protein | 65 | 0.043 | 0.184 | 4.3 |
| 175 | aconitate hydratase, mitochondrial precursor | 81 | 0.041 | 0.082 | 2.0 |
| 208 | lipoma-preferred partner homolog isoform 1 | 126 | 0.063 | 0.178 | 2.8 |
| 214 | glucose-regulated protein precursor (BiP, HSP70) | 112 | 0.111 | 0.254 | 2.3 |
| 222 | E3 SUMO-protein ligase PIAS2 isoform 2 | 68 | 0.032 | 0.128 | 4.0 |
| 229 | propionyl-CoA carboxylase alpha chain, mitochondrial precursor | 144 | 0.030 | 0.063 | 2.1 |
| 241 | albumin precursor | 71 | 0.038 | 0.095 | 2.5 |
| 242 | periostin isoform 3 precursor | 64 | 0.040 | 0.078 | 2.0 |
| 244 | albumin precursor | 101 | 0.030 | 0.124 | 4.1 |
| 245 | albumin precursor | 90 | 0.051 | 0.137 | 2.7 |
| 250 | Heat shock protein 8, heat shock protein 70 cognate | 192 | 0.084 | 0.187 | 2.2 |
| 262 | Sdha protein (Succinate dehydrogenase complex, subunit A, flavoprotein variant) | 138 | 0.039 | 0.094 | 2.4 |
| 265 | lamin B | 136 | 0.044 | 0.097 | 2.2 |
| 273 | Annexin A6 | 155 | 0.043 | 0.162 | 3.8 |
| 280 | Leukotriene A-4 hydrolase | 160 | 0.030 | 0.071 | 2.4 |
| 329 | 60 kDa heat shock protein, mitochondrial | 148 | 0.025 | 0.060 | 2.5 |
| 337 | WD repeat domain 18, isoform CRA_b | 64 | 0.034 | 0.073 | 2.1 |
| 358 | polymerase I and transcript release factor | 85 | 0.072 | 0.140 | 2.0 |
| 384 | vimentin | 146 | 0.034 | 0.074 | 2.1 |
| 412 | Cleavage and polyadenylation factor subunit homolog (S. cerevisiae) | 65 | 0.020 | 0.039 | 2.0 |
| 481 | pyruvate dehydrogenase E1 alpha 1 precursor | 111 | 0.044 | 0.095 | 2.2 |
| 507 | acetyl-Coenzyme A acetyltransferase 1 precursor | 89 | 0.065 | 0.134 | 2.0 |
| 512 | serine (or cysteine) proteinase inhibitor, clade B, member 9 | 79 | 0.019 | 0.176 | 9.1 |
| 545 | eukaryotic translation initiation factor 2, subunit 1 alpha | 128 | 0.011 | 0.028 | 2.5 |
| 562 | alcohol dehydrogenase | 85 | 0.034 | 0.075 | 2.2 |
| 627 | h-2 class I histocompatibility antigen, TLA(B) alpha chain-like | 64 | 0.104 | 0.229 | 2.2 |
| 770 | crystallin, alpha B | 162 | 0.041 | 0.092 | 2.2 |
| 794 | Histone H2B type 1-P | 63 | 0.050 | 0.268 | 5.4 |
| 802 | profilin-1 | 125 | 0.104 | 0.309 | 3.0 |
| 923 | propionyl CoA-carboxylase alpha-subunit | 64 | 0.054 | 0.128 | 2.4 |
| 925 | ATPase, class II, type 9B, isoform CRA_a | 64 | 0.041 | 0.081 | 2.0 |
| 930 | zinc finger protein 271 | 65 | 0.033 | 0.068 | 2.1 |
| 959 | choline dehydrogenase | 65 | 0.023 | 0.058 | 2.6 |
| 962 | sorting and assembly machinery component 50 homolog | 73 | 0.020 | 0.041 | 2.0 |

**
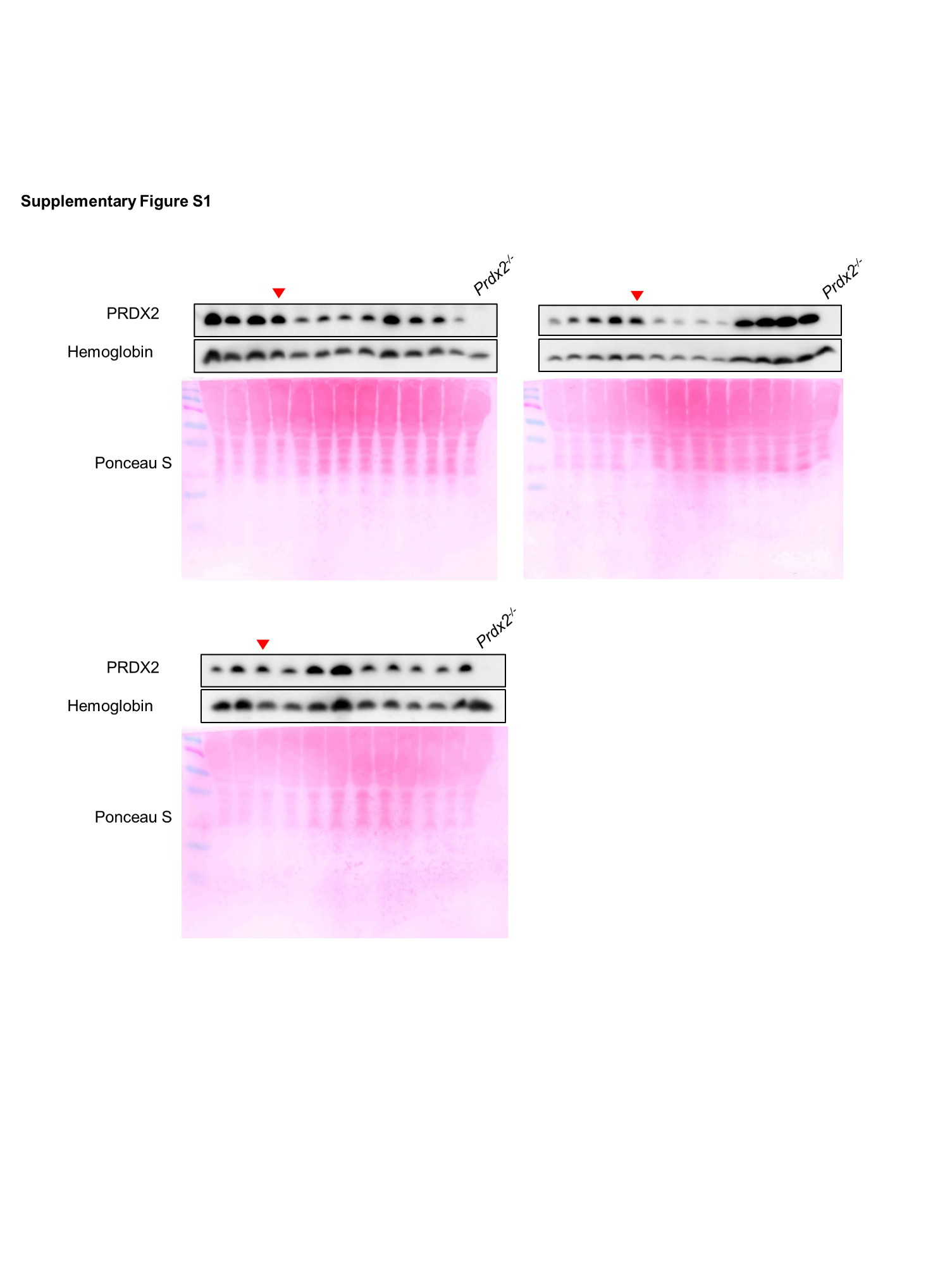
Supplementary Fig. 1.**

**Supplementary Fig. 2.**


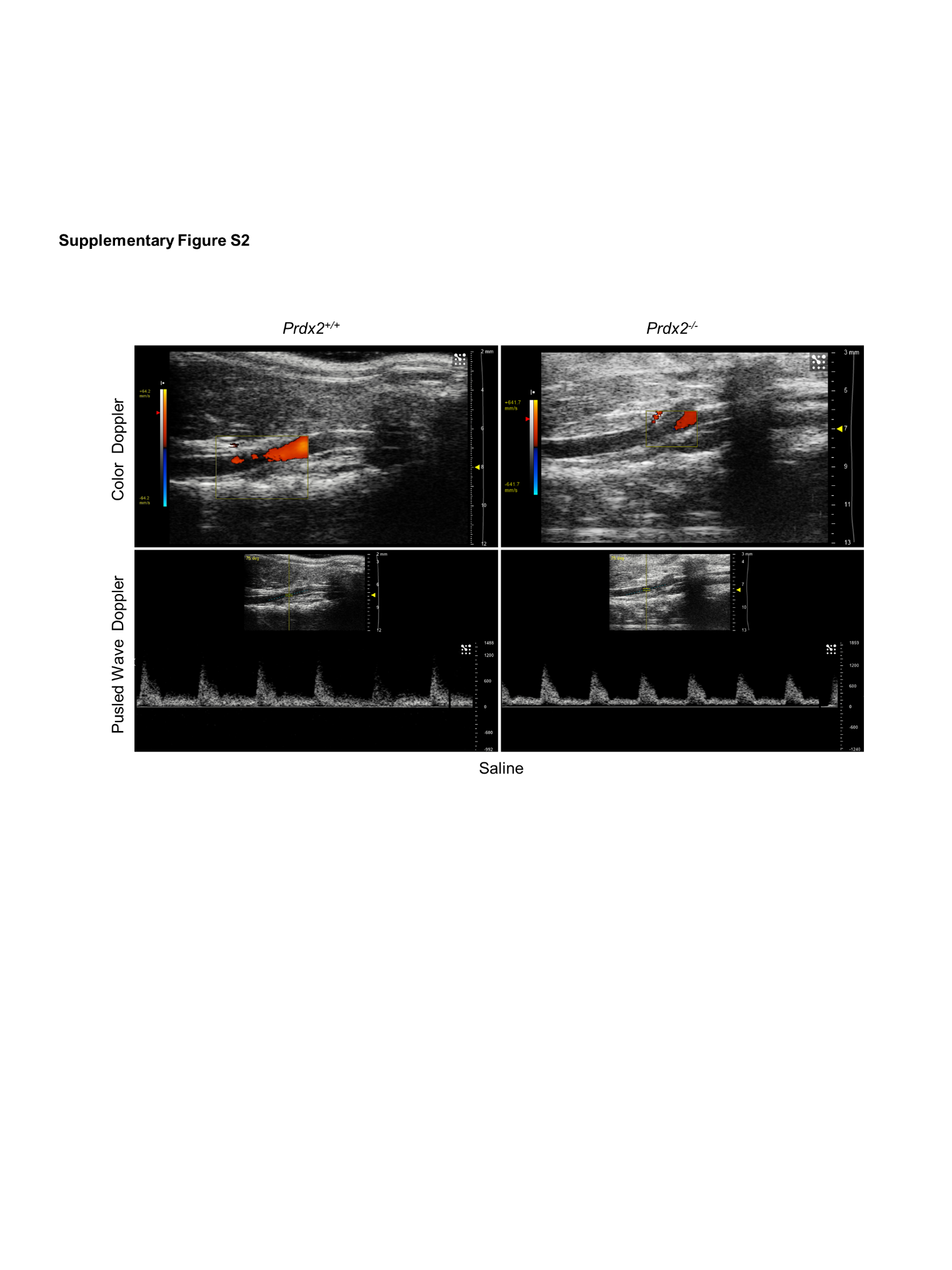


**
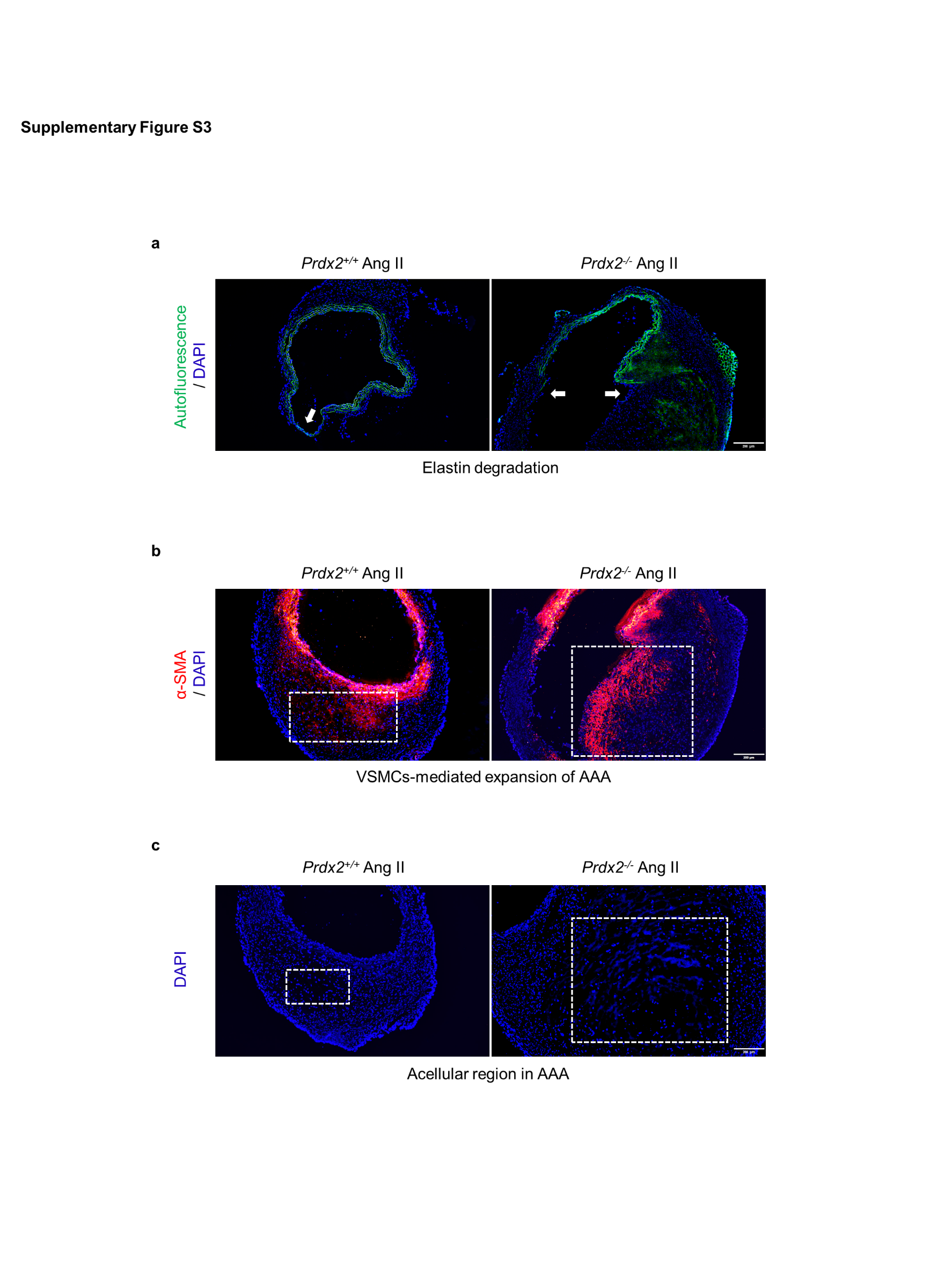
Supplementary Fig. 3.**

**
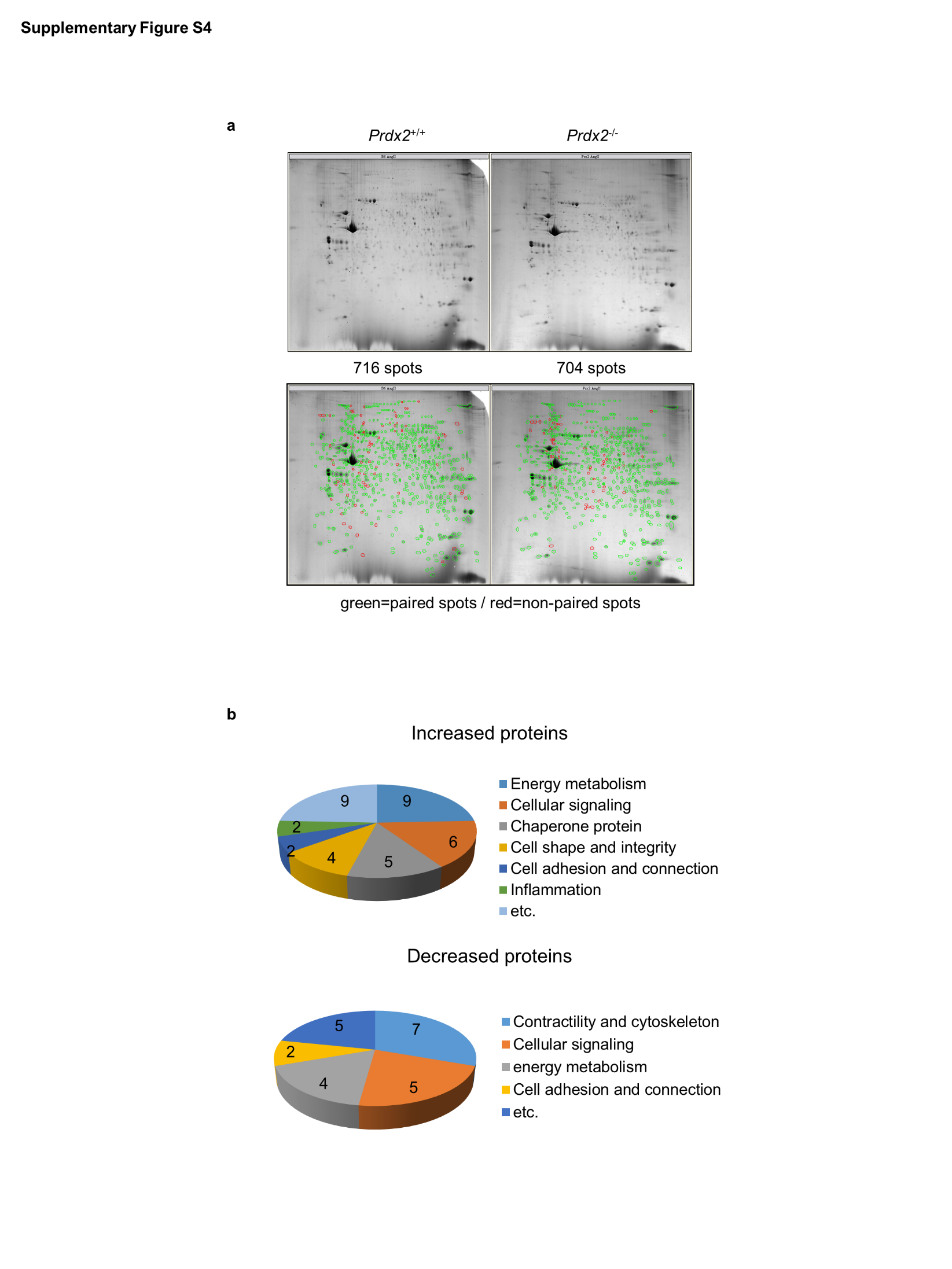
Supplementary Fig. 4.**

**
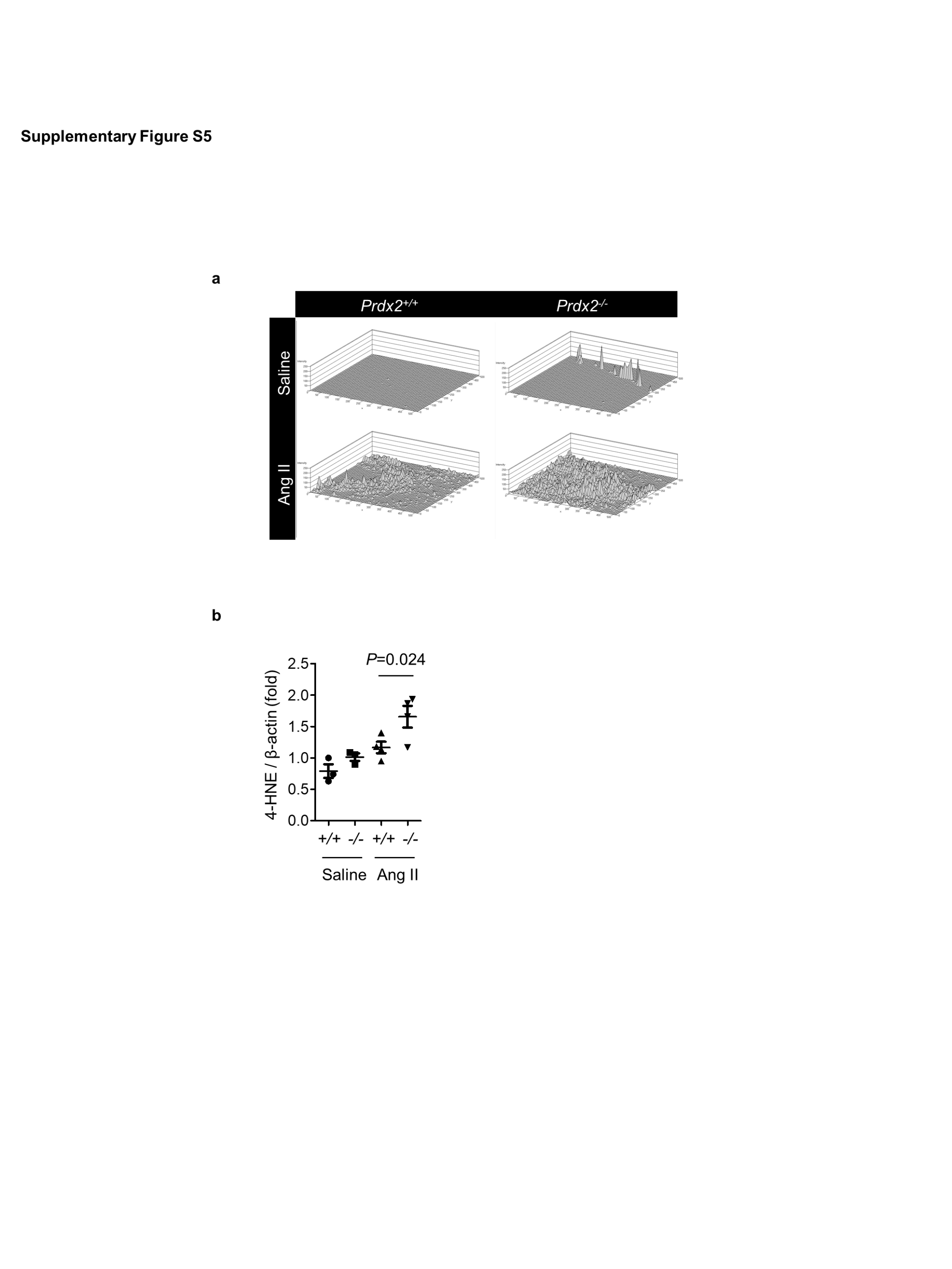
Supplementary Fig. 5.**
